# The Influence of Unconscious Perceptual Processing on Decision-Making: A New Perspective From Cognitive Neuroscience Applied to Generation Z

**DOI:** 10.3389/fpsyg.2020.01728

**Published:** 2020-08-11

**Authors:** Dolores Lucía Sutil-Martín, Juan José Rienda-Gómez

**Affiliations:** ^1^Department of Business Economics, Rey Juan Carlos University, Móstoles, Spain; ^2^Department of Financial Economics and Accounting and Modern Language, Rey Juan Carlos University, Móstoles, Spain

**Keywords:** cognitive neuroscience, unconscious perceptual processing, backward visual masking, generation Z, neuroticism, extroversion, introversion, logistic regression

## Abstract

Cognitive neuroscience and its applied developments have revolutionized marketing. With advances in neuroscientific techniques, marketing has needed to refocus toward understanding issues like the area of the brain that should be stimulated to transform the consumer’s intention to purchase into a real decision, how information is processed when making a decision, and how personality traits affect the purchase decision. Neuroscience has opened the door to the consumer’s brain. For many years, scientists have investigated the role of subliminal messages in marketing, with their findings generating a significant controversy. Many have shown that making sound decisions based on intuition rather than conscious reasoning is more common than previously thought. In fact, many studies have shown that sound intuitive decision-making depends on the association of the subliminal messages of a given situation with the limbic brain structures formed. Scientists have concluded that the brain does not consciously need to know contextual information to learn the value of this information and make the necessary linkages to make productive decisions. In this study, we consider whether unconscious perceptual processing influences decision-making and explore the influence of aspects of personality that are related to unconscious processing, such as the degree of neuroticism, extroversion, and gender of the individual, applied to the demographic cohort Generation Z, distinguishing between whether the stimuli are verbal or pictorial. The backward masking visual paradigm has been used to assess unconscious perceptual processing. To test these processes, a set of ANOVA models and logistic regressions were run where the dependent variable is whether the people perceived the stimuli or not and the independent variables were gender, the form of the stimuli (pictorial or verbal), and the personality traits extroversion, introversion, and neuroticism. The results suggest that verbal stimuli work better than pictorial stimuli, although a possible explanation is that the pictures require modification to be more effective. In the case of verbal stimuli, gender and level of neuroticism are found to be important variables that influence unconscious perceptual decision-making processes. Specifically, a female with a high level of neuroticism shows greater permeability in its unconscious perceptual processes.

## Introduction

Human beings have the potential to perceive all the external stimuli that surround them. Stimuli are processed at different levels: some by conscious perceptual experience, i.e., those we are normally aware of, and others by unconscious perceptual experience, which are received without us having the notion that they are influencing our behavior. This second type of stimulus, namely, subliminal simulation, is what we consider in this paper. The subliminal presentation of the stimuli is based on ensuring that the stimulus is registered by the appropriate sensory system and activates its corresponding representation, but with minimal activation so that the stimulus does not reach consciousness (Smith and McCulloch, 2012).

Demonstrating the existence of unconscious perceptual processes through which stimuli are perceived when subjects are not aware of them has been in the minds of scientists and researchers for decades, generating much controversy. The main areas of research throughout the 20th century have revolved around the acceptability of the method used to establish the absence of conscious perception and the method to evaluate the unconscious perception of the stimulus (Overgaard and Timmermans, 2010; Smith and McCulloch, 2012). With the rise of research in neurology (Neumann and Klotz, 1994; Dehaene et al., 1998; Eimer and Schlaghecken, 1998; Abrams and Greenwald, 2000; Damian, 2001; Abrams et al., 2002; Kunde et al., 2003; Forster, 2004) and neuroscience in the 21st century (Dehaene et al., 2001; Naccache and Dehaene, 2001a, b; Devlin et al., 2004; Nakamura et al., 2005) scientists have converged on the existence of unconscious perceptual processes.

Several paradigms have been used in research on unconscious perceptual processing, including binocular rivalry (Crick and Koch, 2003; Baars, 2005; Mudrik et al., 2010) inattention (Raymond et al., 1992; Shapiro et al., 1997; Driver and Vuilleumier, 2001; Martens et al., 2002; Rusconi et al., 2006) semantic priming (Kouider et al., 2007; Bruno et al., 2020) and visual masking, which is the most commonly used approach. This paradigm (Marcel, 1983) consists of presenting two stimuli in different ways that prevent the first stimulus from being seen. Several strategies are used to prevent the first stimulus from being perceived: varying the intensity of the second stimulus against the first one by presenting both at the same time or presenting the second stimulus immediately after the first one, ensuring that the second stimulus does not allow the first one to be discerned (van Gaal et al., 2010).

From its first use in 1884 by Pierce and Jastrow (1884) in a forced choice discrimination task between two possibilities to the present day through the results of unconscious perceptual processing (Dell’Acqua and Grainger, 1999; Kunde et al., 2005; van Opstal et al., 2005a, b) functional magnetic resonance imaging (fMRI) (Brown and Hagoort, 1993; Lleras and Enns, 2004; Verleger et al., 2004; Goodman et al., 2017; Hakkak et al., 2019) and intracranial registers (Cohen et al., 2000; Henson, 2003) this paradigm ensures that a stimulus reaches the consciousness, fulfilling two conditions proposed by Kouider and Dehaene (2007). The first condition is that the input stimulus has enough strength to cross the global threshold, which can be avoided by degradation of the stimulus or competition with other stimuli, i.e., visual masking. The second is that the stimulus must receive downward amplification by distant neurons, which can be avoided by attracting those neurons to another stimulus or task. In this research, the paradigm that has been used is that of visual masking with different intensity.

Selective attention can also operate dynamically in time [Coull and Nobre (1998) as cited in Pichon et al. (2016)]. Behavioral studies have researched the effect of temporal attention on the perception of visual stimuli flashed quickly in a continuous sequence or briefly presented (Pichon et al., 2016).

Recently, neuropsychologists like Pessiglione (Merikle, 1988; Prévost et al., 2010; Pessiglione et al., 2015; Lopez-Persem et al., 2016) have shown that cases of making sound decisions based on intuition rather than conscious reasoning are more common than previously thought. In fact, they have shown that sound intuitive decision-making basically depends on the association of the subliminal messages of a given situation with the limbic brain structures formed. Krishnan and Trappey (1999) and Theus (1994) reviewed research on subliminal advertising, without considering the role of gender and personality in unconscious processes. Pratkanis and Greenwald (1988) replicated subliminal effects and new models of unconscious processes and abandoned some controversial motivational assumptions of past perspectives. Rosen and Singh (1992) investigated the effect of subliminal sex and death embeds on attention to advertising, change in attitude, behavioral intention, and day-after recall of advertising for two products. No significant effects were indicated in the study at any level.

Synodinos (1988) claimed that stimuli too weak to be detected can affect behavior in powerful ways, finding that when an objective definition was adopted and proper psychophysical methods were followed, there is no support for the effectiveness of undetectable stimuli. An approach was adopted, which used phenomenal awareness as a basis for distinguishing between conscious and unconscious perceptual processes. Trappey (1996) conducted a meta-analysis to demonstrate the ineffectiveness of subliminal advertising in influencing the consumer’s decision between alternatives. However, neither gender nor personality was considered.

Pessiglione et al. (2015) set up visual cues with abstract symbols to evaluate visual perception using “hidden” signals in the abstractions and then ask the participants whether they perceived any difference. If the subjects could not correctly identify the differences, like the introduction of an image of a face (Aguado et al., 2014) then neither could they consciously depict associations between a signal and a result. A second set of experiments was performed using subliminal conditioning using the same procedure involving abstract symbols but relating the results to monetary rewards. The results showed that the rewards and the punishments of subliminal signals guided responses and decisions, including those conditional on abstract signals that the subjects could not consciously perceive. Images obtained through a fMRI scan showed the specific brain circuits related to subliminal conditioning (Pichon et al., 2016). The subliminal fMRI findings are consistent with a view that the automatic activation of affective processes guides evolutionarily advantageous decision-making mechanisms (Panksepp, 2011 as cited in Brooks and Stein, 2014). It can be concluded that the brain does not need to consciously know contextual information to learn the value of this information and make the necessary connections to undertake positive decisions (van Gaal et al., 2010).

To examine personality traits, Eysenck (1994) is followed, which proposed that the extraversion–introversion dimension (extraversion – positive affectivity marked by pronounced engagement with the external world and characterized by high sociability, talkativeness, energy, and assertiveness) is caused by variability in cortical arousal. At low environmental arousal potential, extraverts’ cognitive performance would be lower than that of introverts (Eysenck, 1994 as cited in Mitchell and Kumari, 2016). Besides that, Eysenck’s model treats neuroticism and psychoticism dimensions as independent of extraversion. The model proposes that the neuroticism stability dimension (neuroticism – negative affectivity marked by emotional instability and low tolerance for stress or aversive stimuli and characterized by anxiety, fear, moodiness, worry, envy, frustration, jealousy, and loneliness) is explained by differences in the level of activity primarily in the limbic system. A relationship among unconscious processes, personality traits, and decision-making can be found in many studies (Nga and Yien, 2013; Abadie and Waroquier, 2019; Dell’Orco et al., 2019; Myrica, 2019).

In this study, we also consider whether unconscious perceptual processing influences decision-making. We specifically explore the influence of personality traits that are related to unconscious processing, namely, the degree of neuroticism, extroversion, and introversion as well as the gender of the individual.

## Study Design and Methodology

In this study, the following independent variables were considered: personality (degree of neuroticism, extroversion–introversion), subliminal stimulus type (verbal or pictorial), and gender of individuals. The influence in decision-making was the dependent variable.

### Subjects

The population chosen consists of individuals between the ages of 18 and 25, belonging to the demographic cohort “Generation Z” (Zheng, 2018) living in Madrid, and who have a profile on a social network. The study population was recruited *via* the following advertisement on Twitter: “We need volunteers, aged between 18 and 25 years and living in Madrid to assist in conducting consumer research.” According to the 2019 Annual Report on Social Media in Spain (Iab. Spain, 2019) 85% of people between 16 and 30 years follow at least one “influencer,” “youtuber,” or “instagrammer.” Considering that under-18s need permission from their legal guardians to participate in this research and Generation Z is assumed to comprise people born between 1994 and 2010, it was decided that an age range between 18 and 25 years would provide a representative sample. In addition, people within this cohort are generally more active on social networks, tend to follow several “instagrammers” and “influencers,” own smartphones, tend to follow trends in fashion closely, and are more permeable to unconscious perceptual processes (Fromm and Read, 2018). The participants signed an informed consent prior to their participation. No personal data were used in this research except gender and age. The subjects were coded with an order number.

The experiment was conducted between September and October 2018. A non-experimental, exploratory, correlational, and cross-sectional design was carried out by means of a horizontal networking sampling, a social networking that usually starts with a multiple (although relatively small) number of initial contacts and then uses these to establish links with other research participants (Geddes et al., 2018) *via* social media like Twitter and Instagram. Horizontal networking uses both strong and weak ties to bridge into new social networks, casting the sampling and recruitment net wide rather than deep (Talón-Ballestero et al., 2019). We obtained a large sample of around 390 individuals which, after filtering and debugging of information according to its relevance and representativeness, was reduced to 200 individuals, of whom 100 were men and 100 were women aged between 18 and 25 years.

To test the hypotheses, a set of ANOVA univariate general linear model (GLM) models (Bruno et al., 2020) and logistic regressions were run. The univariate GLM procedure provides regression analysis and analysis of variance for one dependent variable by one or more factors and/or variables. The factor variables divide the population into groups. Using this GLM procedure, we were able to test the null hypotheses about the effects of other variables on the means of various groupings of a single dependent variable. We were also able to investigate interactions between factors as well as the effects of individual factors, some of which may be random (Garson, 2012).

The binary or dichotomous logit model (Hilbe, 2009) allows to model an equation whose result is interpreted as the probability of belonging to the group, coded as 1. Its expression is as follows:

$$\mathrm{Pr}\left(Y_{i}=1\right)=\frac{1}{1+e^{-\left(\alpha +w_{k}\,X_{k\,i}\right)}}=\frac{e^{\alpha +w_{k}\,X_{k\,i}}}{1+e^{\alpha +w_{k}\,X_{k\,i}}}$$

One of the properties of this model is the interpretation of the parameters, where the sign and the value need to be considered. The term *odds* is defined as follows:

$$O\,d\,d\,s=\frac{Prob\left(Y_{i}=1\right)}{1-Prob\left(Y_{i}=1\right)}=e\,x\,p\,\left(\alpha +\sum_{j=1}^{k}w_{j}\,X_{j}\right)$$

The odds ratio is defined from two associated ratios:

$$o\,d\,d\,s-r\,a\,t\,i\,o=\frac{o\,d\,d\,s\,2}{o\,d\,d\,s\,1}=e\,x\,p\,\left(w_{j}\right)$$

The above expression suggests that a coefficient *w*_*j*_ close to 0 – or, equivalently, an odds ratio close to 1 – will mean that changes in the explanatory variables associated with *X*_*i*_ will not have any effect on the dependent variable. As discussed above, we can interpret the coefficients as the change that occurs in the logit term upon a unit increase in the associated explanatory variable.

The Statistical Software package used was SPSS 22.0. The dependent variable (either verbal or pictorial stimulus) behaves in a dichotomous manner, where category 1 is defined as those cases where the stimulus worked and category 0 corresponds to those respondents where the stimulus did not work (Košíková and Pilárik, 2012; Mladenović et al., 2016). The independent variables chosen were gender and the degree of extroversion, introversion, and neuroticism.

### Procedure

From the 200 participants, 10 groups were formed, which ranged in size from 15 to 25 people, eight of which were to be subject to experimental conditions, with two control groups. In the experimental groups, 50% of subjects were female and 50% were male. In the two control groups, the proportion of females was 60% against 40% for males. The assignment to each group was random.

Once divided, each group went into a room where they completed a personality questionnaire, the Eysenck Personality Inventory Form B for adults (EPI-B), to evaluate their degree of introversion versus extroversion and to grade their extent of neuroticism.

**FIGURE 1:**
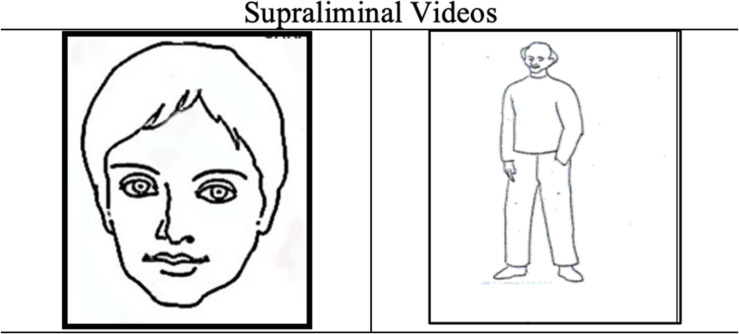
Human face and human figure videos.

After completing the questionnaire, two videos were presented to each group: one depicting a human face, designed to equally represent masculine and feminine features and the other a human figure, a male in a neutral pose with his hands in his pockets (Figure 1).

Each group was given the following instructions before viewing the videos:

“Now you will see two videos, please stay alert because after each video you will need to write on the card the following: when you see a human face, you will have to decide whether this is a man or a woman, and note this on the card; when you see a person, you will have to decide whether that person is brave or cowardly and note this on the card. Thank you very much for your cooperation.”

The order of the presentation of the video was altered such that five out of the 10 groups (four experimental and one control) saw the video of the human face first, while the other five groups (four experimental and one control) saw the video of the human figure first.

### Material

#### Personality Test

We used the EPI-B Spanish version TEA 1973 to evaluate the dimensions of introversion–extroversion and neuroticism. The use of this questionnaire for this exercise is supported by the findings of Mitchell and Kumari (2016) who corroborated Eysenck’s proposals for the biological model of personality and found that extraversion and neuroticism relate meaningfully to the functioning and the structure of various cortical and limbic brain regions. Their analysis demonstrated a robust relationship between neuroticism and the functioning of several emotion processing networks in the brain, especially in the presence of negative stimuli. Close links were found in regions of the brain involved in emotion regulation, depression, and anxiety as well as several sub-cortical and limbic regions.

A total of 10 videos were made using Adobe Premier. The duration of each video was 12 s. For each video, there was a control condition and two experimental conditions.

#### Video 1 – Human Face

**FIGURE 2:**
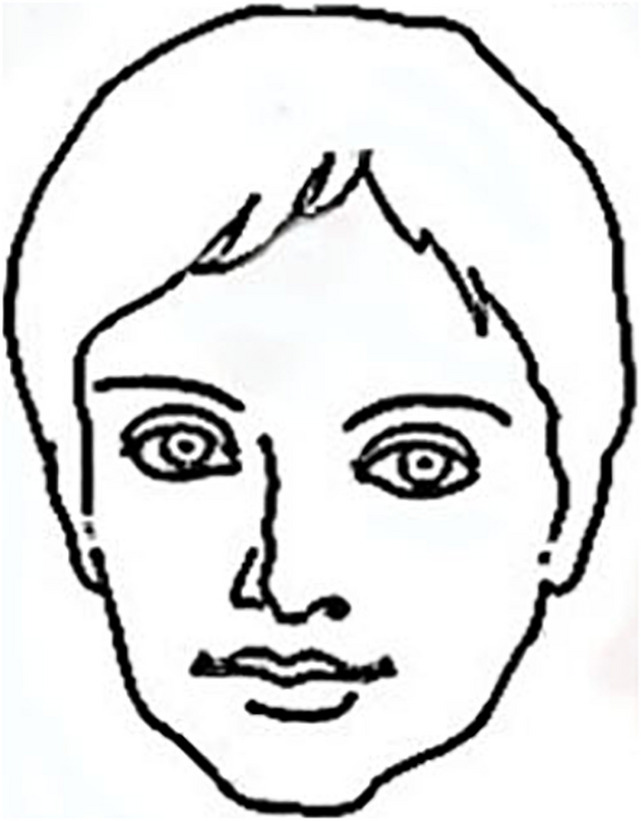
Human face.

Control condition: A human face appears for 12 s (Figure 2).

**FIGURE 3:**
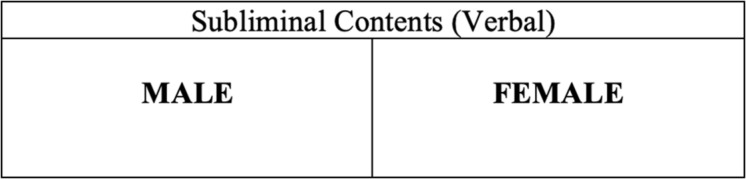
Verbal subliminal stimulus. Male – Female.

Experimental condition 1: A 12-s video of the human face is shown. Every 3 s, a frame is interspersed with the word MALE for 10 ms. This frame is repeated three times (Figure 3).

Experimental condition 2: The same as in experimental condition 1, except that the word FEMALE is used (Figure 3).

#### Video 2 – Human Figure

**FIGURE 4:**
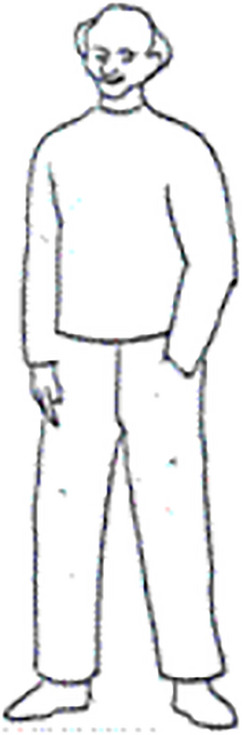
Human figure.

Control condition: A human figure appears for 12 s (Figure 4).

**FIGURE 5:**
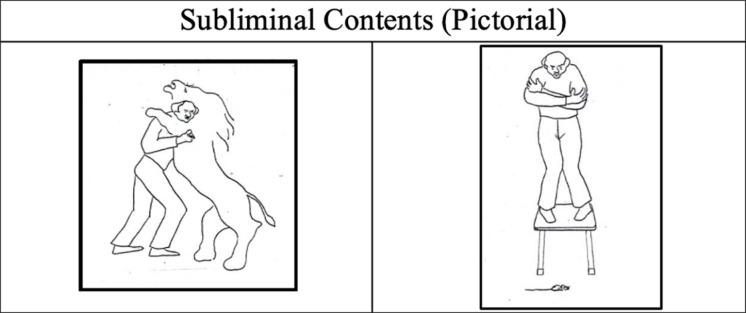
Pictorial subliminal stimulus. Brave – Coward.

Experimental condition 1: A 12-s video of the human figure is shown. Every 3 s, a frame is interspersed with the scene of the same human figure climbing up a stool in front of a tiny frightened mouse. This frame is repeated three times (Figure 5).

Experimental condition 2: The same as in experimental condition 1 except that the interspersed scene was of the same human figure struggling fiercely against a lion (Figure 5).

### Experimental Conditions

Each group was shown two videos (each ran for 12 s), the order of which was altered. The choice of the groups was random. The 10 groups differed as shown in Table 1:

Group 1 was first shown the video containing the human figure with a brave experimental condition and then the second video containing the human face with the male experimental condition.
Group 2 was first shown the video containing the human figure with a cowardly experimental condition and then the video containing the human face with the female experimental condition.
Group 3 was first shown the video containing the human face with the male experimental condition and then the video containing the human figure with a brave experimental condition.
Group 4 was first shown the video containing the human face with the female experimental condition and then the video containing the human figure with a cowardly experimental condition.
Group 5 was first shown the video containing the human figure with the brave experimental condition and then the video containing the human face with the female experimental condition.
Group 6 was first shown the video containing the human figure with the cowardly experimental condition and then the video containing the human face with the male experimental condition.
Group 7 was first shown the video containing the human face with the female experimental condition and then the video containing the human figure with the cowardly experimental condition.
Group 8 was first shown the video containing the human face with the male experimental condition and then the video containing the human figure with the cowardly experimental condition.
Group 9 was first shown the control video containing the human figure and then the control video containing the human face.
Group 10 was first shown the control video containing the human face and then the control video containing the human figure.

**TABLE 1:**
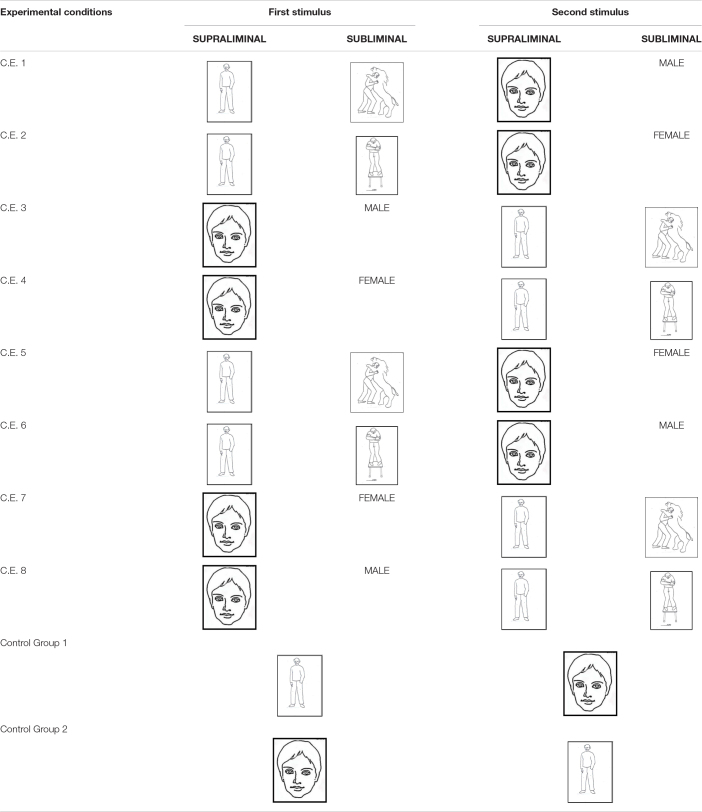
Experimental and control group stimuli.

The videos and stimuli were shown in different orders to evaluate if the order was important for unconscious process.

### Hypotheses

The expansion of cognitive neurosciences and developments in imaging radio-diagnosis techniques, traditionally used in medicine, has driven their use in areas as diverse as marketing and consumer behavior. Knowing how the human brain works, which lobes are activated, and its activity in the face of certain stimuli has allowed a complete theory about human behavior to develop (Opris et al., 2020).

To test the propositions, we divided the sample into control and experimental groups and formulated the first hypothesis as follows:

Hypothesis (H_1_): *The percentage of people who perceive the verbal or pictorial stimuli for the experimental group is greater than for the control group.*

To test whether gender is a factor that can discriminate in the perception of stimulus, we formulate the following hypothesis:

Hypothesis (H_2_): *Gender is a determinant variable for verbal or pictorial stimuli responses.*

To determine if personality traits have influence in stimuli acquisition, we formulated these hypotheses:

Hypothesis (H_3_): *Personality traits are associated with the pictorial stimulus.*

Sub-hypotheses:

Hypothesis (H_3__A_): *Neuroticism as a personality trait is associated with pictorial stimulus.*

Hypothesis (H_3__B_): *Extroversion as a personality trait is associated with pictorial stimulus.*

Hypothesis (H_3__C_): *Introversion as a personality trait is associated with pictorial stimulus.*

Hypothesis (H_4_): *Personality traits are associated with the verbal stimulus.*

Sub-hypotheses:

Hypothesis (H_4A_): *Neuroticism as a personality trait is associated with verbal stimulus.*

Hypothesis (H_4B_): *Extroversion as a personality trait is associated with verbal stimulus.*

Hypothesis (H_4C_): *Introversion as a personality trait is associated with verbal stimulus.*

Although an objective of this study is to establish whether pictorial and verbal stimuli work in the same way, the research also aims to verify that personality and gender traits are decisive for one of these stimuli.

## Main Findings

The data analysis was conducted in stages. In the first stage, a descriptive analysis of the sample was undertaken to determine the proportions of individuals by gender, the average percentiles for personality variables (degree of neuroticism and extroversion–introversion), and the degree of response to the stimuli (see variable code in Table 2). The order in which the stimuli were shown was not found to be important for the unconscious processes.

**TABLE 2:** Summary of variable codes.

Variables	
PC_N / PC_N_C	Neuroticism
PC_E / PC_E_C	Extroversion
PC_I / PC_I_C	Introversion
Cow_Brav / Cow_Brav_C	Stimulus coward–brave
Male_Female / Male_Female_C	Stimulus male–female
VR_CV / VR_MF	True stimuli shown

*“C” after the variable names indicates the control group.*

Table 3 provides the summary of the descriptive statistics for the control group. For the gender variable, 81.82% of men were included in this control group. According to their answers, 93.94% of the respondents thought that the human face was female (the variable was coded “0” as female and “1” as male), and just 18.18% thought that the picture was of a brave man (“0” as a coward and “1” as a brave man). As for their personality traits, the observed percentiles of neuroticism, extroversion, and introversion were 56.93, 73.96, and 61.51, respectively. The personality trait extroversion–introversion was divided into two different variables, and the introversion variable was re-scaled to be measured in the same sense as the extroversion variable. With the re-scaling, the higher percentile of the variable, the more introverted the person.

**TABLE 3:** Summary descriptive statistics of the control group.

	N	Mean	St. Dev.
Gender	33	0.8182	0.39167
Male_Female	33	0.9394	0.24231
Cow_Brav	33	0.1818	0.39167
PC_N_C	33	56.9394	23.92167
PC_E_C	33	73.9697	20.66774
PC_I_C	33	61.5152	28.34180

Table 4 shows the same variables as for Table 3 but for the experimental groups. The percentage of male and females who participated in these groups was 50%. Around three-quarters (75.32%) of the respondents thought that the human face was female, while 34.42% considered the picture to be of a brave man. The percentiles of neuroticism, extroversion, and introversion were 55.36, 68.39, and 61.01, respectively. The results of the difference of proportion tests are shown in Tables 5, 6. These show that there are no statistically significant differences in the proportion of people responding to the subliminal message of the human figure (pictorial stimulus) at 5% significance level (*p* > 0.05, where *p* means test *p*-value). By contrast, when focusing on the human face subliminal message (verbal stimulus), the difference of proportion test shows statistically significant differences between the response of the control and the experimental groups (*p* < 0.05). This is an important finding as it shows that the human face subliminal messaging works.

**TABLE 4:** Summary descriptive statistics of the experimental group.

	N	Mean	St. Dev.
Gender	154	0.5000	0.50163
Male_Female	154	0.7532	0.43253
Cow_Brav	154	0.3442	0.47664
PC_N	155	55.3613	25.32872
PC_E	155	68.3871	20.95555
PC_I	155	61.0129	26.04042

**TABLE 5:** Difference of proportions: Cow_Brav.

Variable	N	Sample proportion
Cow_Brav_C	33	0.181818
Cow_Brav	307	0.345277
Difference = p (Cow_Brav_C) – p (Cow_Brav)		
Estimate for difference	−0.163459	
95% CI for difference	(−0.305394, −0.0215237)	
Test for difference = 0 (vs. not = 0):	*Z* = −2.26 *P*-Value = 0.024*	
Fisher’s exact test: *P*-Value	0.078*	

Sig. Code: * 0.1.

**TABLE 6:** Difference of proportions: Male_Female.

Variable	N	Sample proportion
Male_Female_C	33	0.939494
Male_Female	308	0.594156
Difference = p(Male_Female_C)-p(Male_Female)		
Estimate for difference	0.345238	
95% CI for difference	(0.247080, 0.443396)	
Test for difference = 0 (vs. not = 0):	*Z* = 6.89 *P*-Value = 0.000***	
Fisher’s exact test: *P*-Value	0.000***	

Sig. Code: *** 0.001.

If we compare the results of the mean values in Tables 3, 4, we can see that the values of neuroticism, extroversion, and introversion variables are very similar, both for the control groups and for the experimental groups. A contrast of mean difference was carried out, in which the *p*-values were significant at all accepted levels, which allows us to conclude that there are no differences in the percentiles of the variables described above between the control and the experiment groups. This finding allows us to conclude that there is no bias in the assignment of individuals to the reference group, so the individuals are well distributed in both the control and the experimental groups.

In the second phase, the variance for the two types of stimuli (verbal and pictorial) was modeled (as shown in Tables 7, 8). In the first model, which considered the pictorial stimulus type (coward–brave), the gender of individuals was detected as a variable influencing decision-making (*p* < 0.05). However, neither the personality trait variables nor the type of unconscious perceptual processing was found to be significant (*p* > 0.05). In the second model, which considered the verbal stimulus type (male–female), the degree of neuroticism and the gender of the individual were detected as influential variables in decision-making (*p* < 0.05), but neither extroversion nor introversion.

**TABLE 7:** ANOVA univariate model: Cow_Brav.

Origin	Type III sum of squares	df	Squared mean	F	Sig.
Adjusted model	1.763	3	0.588	2.663	0.050*
Intercept	2.758	1	2.758	12.501	0.001***
PC_N	0.562	1	0.562	2.545	0.113
PC_I	0.121	1	0.121	0.551	0.459
Gender	1.363	1	1.363	6.178	0.014**
Error	32.878	149	0.221		
Total	53.000	153			

R-squared = 0.051 (R-squared adjusted = 0.032) Dependent Variable: Cow_Brav. Sig. Codes: *** 0.001, ** 0.01, * 0.1.

**TABLE 8:** ANOVA univariate model: Male_Female.

Origin	Type III sum of squares	df	Squared mean	F	Sig.
Adjusted model	22.551	3	7.517	73.696	0.000***
Intercept	0.232	1	0.232	2.271	0.134
PC_N	1.440	1	1.440	14.122	0.000***
PC_E	0.022	1	0.022	0.215	0.644
Gender	21.003	1	21.003	205.916	0.000***
Error	15.300	150	0.102		
Total	67.000	154			

R-squared = 0.596 (R-squared adjusted = 0.588) Dependent Variable: Male_Female. Sig. Code: *** 0.001.

To carry out this analysis, personality traits were converted into factors, coded as 0 when there is an absence of personality trait factor.

In the third phase, a binary logit model was used to quantify the variables that affect the propensity to influence unconscious processing in decision-making (Tables 9, 10). A logit model is a kind of a regression model which is more appropriate because the dependent variable is dichotomous. The participants watched two videos (human figure and human face). The results of the first video (human figure) support that gender is a significant variable (*p* < 0.05), but neither personality traits nor assimilation of the stimulus were found to be significant (*p* > 0.05). An odds ratio estimate of 2.548 implies that a male is twice more likely to be influenced by the pictorial stimulus than a female, but no influence of personality traits was detected.

**TABLE 9:** Binary logit model: Cow_Brav.

	Coefficients	Statistic error	Wald	df	Sig.	Exp(B)
Gender	0.935	0.372	6.307	1	0.012*	2.548
PC_N	−0.009	0.007	1.590	1	0.207	0.991
PC_E	0.012	0.009	1.695	1	0.193	1.012
PC_I	0.004	0.007	0.417	1	0.518	1.004
VR_CV	−0.169	0.374	0.204	1	0.652	0.845
Intercept	−1.597	0.994	2.582	1	0.108	0.202

Sig. Code: * 0.1.

**TABLE 10:** Binary logit model : Male_Female.

	Coefficients	Statistic error	Wald	df	Sig.	Exp(B)
Gender	−4.927	0.802	37.769	1	0.000***	0.007
PC_N	0.052	0.014	13.801	1	0.000***	1.053
PC_E	0.014	0.015	0.810	1	0.368	1.014
PC_I	−0.013	0.013	1.164	1	0.281	0.987
VR_MF	2.089	0.616	11.498	1	0.001***	8.079
Intercept	−2.455	1.581	2.410	1	0.121	0.086

Sig. Code: *** 0.001.

For the second video (verbal stimulus), the variables gender, assimilation of the stimulus, and degree of neuroticism are significant (*p* < 0.05), meaning that an influence on the unconscious perceptual stimulus is more likely for females. Being influenced by verbal stimuli reduces by 0.7% for males. An increase in one percentile unit of the level of neuroticism increases the perception of verbal subliminal stimulus by 5%. In terms of the perception of true stimulus, being a woman and having high levels of neuroticism represents twice as likely to assimilate such stimulus against the rest of the individuals.

## Discussion

To verify the applicability of these new paradigms, many studies are conducted by dividing the sample into control group and experimental group (Takemura, 2019; Kalkova et al., 2020). If the proportion of individuals in the experimental group who perceive the stimulus produced by the subliminal message is statistically superior to the proportion of individuals in the control group who can respond by chance, then we can say that the stimulus works.

If the responses are different by groups and one of them is more prone to be influenced by subliminal messages, this means that the subliminal messages work. According to our hypothesis, we can conclude that *H*_1_ can be accepted.

Several studies show that consumer decision-making and behavior are determined by different aspects but are clearly differentiated by gender (Lin et al., 2019). Consistent with the Maslow pyramid, the need and motivation to purchase depends on impulsivity, acceptance, and prior conditioning of beliefs and attitudes as well as personality traits and gender.

As Tables 7, 8 show, for both verbal and pictorial stimuli [as seen in Bruno et al. (2020)] the gender variable is determinant in explaining unconscious processes. In addition, for verbal stimuli, variables such as gender and the level of neuroticism appear important to perceive the subliminal stimulus. In this case, we can accept the *H*_2_ hypothesis.

Finally, on personality traits, Bustin et al. (2012) and Mitchell and Kumari (2016) have studied the cognitive neuroscience of personality through fMRI and the brain regions that are activated in the face of attitudes of extroversion, introversion, and neuroticism, based on Eysenck’s biological model (Eysenck, 1994) as well as how unconscious processes can be modulated from subliminal reward signals. In this case, this study aims to shed more evidence to determine whether personality traits influence the perception of subliminal stimuli.

For the video of the human figure (pictorial stimulus), the results differ somewhat. While gender is significant, meaning that females again better perceive the unconscious stimulus, this does not have a measurable effect on subliminal induced response. While they receive the stimulus, they fail to give the right answer (Bruno et al., 2020). As the results show, hypothesis *H*_3_ and all its sub-hypotheses (*H*_3A_, *H*_3B_, and *H*_3C_) can be rejected where personality traits do not determine the perception of the subliminal message (see Tables 9, 10).

In view of this result, we considered the reasons why this stimulus has failed to influence decision-making.

We analyzed different experimental groups alongside the control groups through a statistical analysis of the difference of response ratios. For the human figure (coward–brave), the results were not statistically significant at the 1% level against the control group, whereas the results were statistically significant for the human face (male–female) (Amihai et al., 2011; Aguado et al., 2014; Bruno et al., 2020).

For the video of the human face (verbal stimulus), the results are stronger. Both gender and the level of neuroticism turn out to be relevant variables when perceiving the stimulus in an unconscious decision-making process (Aguado et al., 2014).

Females are far more influenced by unconsciously perceived stimuli in decision-making. In addition, females have a higher level of perceptual unconsciousness than males, resulting in them assimilating to a greater degree the unconscious stimulus and its influence on decision-making. The degree of neuroticism is a condition that favored the assimilation of subliminal stimuli, that is, those with high levels of neuroticism are more likely to better assimilate the stimulus. As a result, people with emotional liability and hyperactivity, that is, being emotionally hypersensitive, are more influenced by unconscious stimulation than those with low levels of neuroticism. Following this finding, hypothesis *H*_4_ can be partially accepted. Hypothesis *H*_4A_ can be accepted, but both *H*_4B_ and *H*_4C_ can be rejected.

## Conclusion

This paper was motivated by the gap detected in the literature regarding the role of gender and personality traits in response to subliminal messages and in unconscious processes. Previous studies have followed two broad approaches: the first with personality variables and unconscious decision-making processes, subliminal messages, and visual masking or semantic priming techniques and the second with verbal and pictorial messages, using the same techniques, but without considering the personality characteristics or the gender of individuals.

By contrast, this study has dealt simultaneously with different subliminal messages, both verbal and pictorial, personality traits, and gender. For decision-making purposes, it is important to know if males or females are more likely to be influenced by subliminal messages and whether these have measurable effects on their behavior. Furthermore, different personality traits can affect this influence. Therefore, it would be advisable for digital marketers like “influencers” and “instagrammers” to become aware of these direct specific actions aimed at certain objectives to strengthen loyalty in their brands and make marketing campaigns more effective.

In view of the results obtained, it can be confirmed, unlike in previous studies where techniques such as the use of fMRI were not applied, that unconsciously processed information can influence decision-making. The verbal stimulus and levels of neuroticism show statistically significant impacts in measuring the effect of behavior on the unconscious decision-making process. Neither extroversion nor introversion was shown to be relevant for this unconscious decision-making processes, under either of the two stimuli. Under the experimental conditions, the subliminal verbal stimulus was more effective than the pictorial stimulus, as recent research has shown. In an attempt to investigate this result and, in particular, the bias toward associating the human figure (pictorial stimulus) as a coward in the responses of the experimental groups, we asked if there was a feature of the figure that made them think it was cowardly. The participants answered that the human figure had a hand in his pocket, which signified that he was hiding something and therefore was a coward. This response must be considered in a subsequent investigation.

Gender has a measurable effect for both verbal and pictorial stimuli. However, for the pictorial stimulus, we can only conclude that the behavior is different for males and females, observing that being male increases the probability of stating that the individual in the human figure is brave but without reflecting statistical significance. For the verbal stimulus, the gender variable is highly significant, in addition to representing a measurable effect together with the actual stimulus.

This paper presents several limitations that will guide the development of future research. The results are limited to Spanish Twitter users and the way they follow “influencers,” “youtubers,” and “instagrammers.” Therefore, the evidence shown in this paper cannot be generalized to different social media or geographical contexts. Moreover, it is necessary to develop a broader study considering other personality traits, following a model different from Eysenck’s theory, and adding other variables like extended age range, social status, and economic resources. Another limitation is the way the videos were presented and the time between subliminal messages. Due to the technical characteristics of the devices used, the presentation time did not allow us to decrease the presentation time of the subliminal stimuli, which might have influenced the direction of the response.

Given the results of this work, two different lines of further research are being carried out. In the first, we are adapting a newly developed model of personality traits, and in the second, new complementary technical devices are being considered, like eye-tracking, blood pressure, galvanic skin response, and 3D video.

## Data Availability Statement

The raw data supporting the conclusions of this article will be made available by the authors, without undue reservation.

## Ethics Statement

Ethical approval was not provided for this study on human participants because at the request of the research team, the Research Ethics Committee at King Juan Carlos University was consulted and we were verbally confirmed that, in accordance with our local legislation and institutional requirements our study was exempt from ethical approval, for its specific characteristics. The patients/participants provided their written informed consent to participate in this study.

## Author Contributions

D-SM and JR-G contributed to the introduction, study design and methodology, main findings, and conclusion. Both authors contributed to the article and approved the submitted version.

## Conflict of Interest

The authors declare that the research was conducted in the absence of any commercial or financial relationships that could be construed as a potential conflict of interest.
