# Corrigendum: The Influence of Unconscious Perceptual Processing on Decision-Making: A New Perspective From Cognitive Neuroscience Applied to Generation Z

**DOI:** 10.3389/fpsyg.2021.752308

**Published:** 2021-09-02

**Authors:** Dolores Lucía Sutil-Martín, Juan José Rienda-Gómez

**Affiliations:** ^1^Department of Business Economics, Rey Juan Carlos University, Móstoles, Spain; ^2^Department of Financial Economics and Accounting and Modern Language, Rey Juan Carlos University, Móstoles, Spain

**Keywords:** cognitive neuroscience, unconscious perceptual processing, backward visual masking, generation Z, neuroticism, extroversion, introversion, logistic regression

In the original article, there was a mistake in *Table 6**: Difference of proportions: Male_Female* as published. There are two different typographical errors. The right value of the sample proportion for Male_Female_C is 0.939394, and the sample size is 33. The corrected *Table 6**: Difference of proportions: Male_Female* appears below.

**Table 6:** Difference of proportions: Male_Female.

**Variable**	**N**	**Sample proportion**
Male_Female_C	33	0.939494
Male_Female	308	0.594156
Difference = p(Male_Female_C)-p		
(Male_Female)		
Estimate for difference	0.345238	
95% CI for difference	(0.247080, 0.443396)	
Test for difference = 0 (vs. not = 0):	Z = 6.89 P-Value = 0.000^***^	
Fisher's exact test: *P*-value	0.000^***^	

*Sig. Codes*:

*** *0.001*.

The authors apologize for this error and state that this does not change the scientific conclusions of the article in any way. The original article has been updated.

## Publisher's Note

All claims expressed in this article are solely those of the authors and do not necessarily represent those of their affiliated organizations, or those of the publisher, the editors and the reviewers. Any product that may be evaluated in this article, or claim that may be made by its manufacturer, is not guaranteed or endorsed by the publisher.

